# Maternal mental health matters: Indicators for perinatal mental health—A scoping review

**DOI:** 10.1371/journal.pone.0317998

**Published:** 2025-01-27

**Authors:** Elly Layton, Alexandra Roddy Mitchell, Elissa Kennedy, Allisyn C. Moran, Francesca Palestra, Neerja Chowdhary, Shanon McNab, Caroline S. E. Homer

**Affiliations:** 1 Maternal, Child and Adolescent Health Program, Burnet Institute, Melbourne, Australia; 2 Department of Obstetrics, Gynaecology and Newborn Health, University of Melbourne, Melbourne, Australia; 3 Murdoch Children’s Research Institute, Melbourne, Australia; 4 Department of Maternal, Newborn, Child and Adolescent Health and Ageing, World Health Organization, Geneva, Switzerland; 5 Department of Mental Health and Substance Use, World Health Organization, Geneva, Switzerland; 6 MOMENTUM Country and Global Leadership, Jhpiego, Washington, District of Columbia, United States of America; School of Nursing Sao Joao de Deus, Evora University, PORTUGAL

## Abstract

Perinatal mental health disorders are a significant contributor to morbidity and mortality in childbearing women. The World Health Organization recommends all women be screened for mental health disorders postnatally and have diagnostic and management services available. There are, however, currently no global indicators in use which measure the status and progress of perinatal mental health. The aim of this scoping review was to identify existing perinatal mental health indicators and propose a core set which could be used at a global level. We used the Global Perinatal Mental Health Theory of Change as the conceptual framework. We found 25 indicators for PMH aligned with the Global Perinatal Mental Health Theory of Change, which were condensed to form a core set of nine indicators These core indicators include the proportion of women with depression, anxiety, post-traumatic stress disorder, psychosis, or adjustment disorders in the perinatal period; the proportion of women screened for these services; the proportion who have access to services following a positive diagnosis; and, the proportion of healthcare providers trained to provide mental health care. This review forms part of the foundational work for the development of a global monitoring framework which would be able to monitor progress towards the provision of universal high quality perinatal mental health care.

## Introduction

Perinatal mental health (PMH) disorders are among the most common morbidities of the perinatal period [1, 2]. The perinatal period represents a unique time in a woman’s life when there are significant physical and social changes [3]. During this time, women are at higher risk of new onset of mental health disorders, or recurrence of pre-existing mental health conditions [4, 5]. PMH disorders are associated with morbidity and mortality [4]. In women, they are associated with increased obstetric complications, reduced quality of life, substance use, and suicide [6–8]. They increase the likelihood of women experiencing poverty, physical health complications and intimate partner violence [8]. Further to this, PMH disorders can increase the risk of preterm birth, and poor fetal and infant growth and may impact the mother-infant bond [9, 10]. In children, caregiver PMH disorders can lead to poor neurodevelopmental outcomes and an increased risk of mental health disorders in adolescence [11, 12]. At the societal level, PMH disorders add a significant social and economic burden [4]. Globally, it is estimated that the prevalence of PMH disorders is 10–20% [10, 13], however, the majority of research has been performed in high-income countries [HIC], and rates are thought to be increased in low- and middle-income countries (LMIC) [11, 14].

The past two decades have seen a growing focus on the importance of PMH, however there are currently no global indicators that can measure the status or progress towards universal access to high-quality PMH care [10, 15, 16]. The prevention, identification, and treatment of women with PMH disorders is now included in multiple World Health Organization [WHO] guidelines for maternal and child health [16–18]. The Sustainable Development Goals also include the promotion of maternal mental health [19]. However, there is significant variation in policy, guidelines, service availability, screening tools, and implementation approaches worldwide [4, 9].

Indicators are instruments with the objectives of improving population health and reducing inequalities, and are a vital means of informing decision-making [20]. A monitoring framework with key indicators would enable consistent measurement of the status of PMH globally. It would inform actions aimed at improving the prevention, care, and treatment of PMH disorders, and facilitate monitoring and evaluation of progress towards specified targets. Therefore, the aim of this review is to identify existing PMH indicators published in the literature and create a core set of indicators to inform future development of a monitoring framework which can be used to measure PMH globally.

## Methods

A protocol including the scope and aims of this research as well as all subsequent findings was reviewed and refined in consultation with: WHO Department of Maternal, Newborn, Child, Adolescent Health and Ageing; WHO Mother and Newborn Information Tracking Outcomes and Results (MoNITOR) group; WHO Department of Mental Health and Substance Use; and members from the USAID MOMENTUM Country and Global Leadership PMH Community of Practice (S1 Appendix). Findings were reported using the Preferred Reporting Items for Systematic Reviews and Meta-Analyses Extension for Scoping Reviews (PRISMA-ScR) checklist (S1 Table) [21].

### Conceptual framework

The Global PMH Theory of Change (PMH ToC), supported by USAID’s MOMENTUM Country and Global Leadership (MOMENTUM) project, was developed following MOMENTUM’s landscape analysis on the burden of PMH disorders [22, 23]. The PMH ToC offers a framework to guide global PMH development towards the goal of widely accessible, high-quality PMH prevention, care and treatment services. It is based on a social ecological model that places the woman at the centre and incorporates interventions and outcomes across five domains: individual, interpersonal relationships, community, service delivery ecosystem and policy landscape. The impact includes improved PMH and wellbeing for all women [23]. The WHO Guide for Integration of Perinatal Mental Health includes methods of integrating perinatal mental health into routine maternal and child health services [24]. Based on this guideline and informed by findings of the scoping review, we adapted the PMH ToC to focus on individual outcomes and service delivery ecosystem interventions. This review only includes indicators which align with the guideline and does not include risk factors for the development of PMH disorders as included in the PMH ToC.

### Search strategy and screening processes

The search strategy combined terms related to the perinatal period, mental health, and indicator (S2 Table). We performed the systematic search in three databases (MEDLINE, Embase and PsycINFO). Studies published between 1 January 2000 and 4 October 2024, were included. This time frame was selected to ensure contemporary studies were included. Studies were screened independently by two members of the research team (EL & ARM]) with any discrepancies on the inclusion/exclusion resolved by discussion. The process was managed using Covidence software [25]. We also extended our search to non-peer-reviewed material, thematically searching the websites of WHO, OECD, UNICEF Multiple Indicator Cluster Surveys (MICS), and The Demographic and Health Survey [DHS] for databases and reports which may include indicators for PMH [15, 26–28]. We also searched Governmental websites from English speaking countries which were referenced in peer reviewed sources and therefore likely to contain indicators (United Kingdom, Australia, United States of America). For each of these resources, available indicator dashboards were systemically reviewed for relevant indicators and the keyword ‘perinatal mental health’ was searched. Finally, we reached out to researchers working in this field for any additional relevant materials, which did not result in extra resources.

### Inclusion and exclusion criteria

The definition of PMH used in this review was taken from the landscape analysis and PMH ToC, which define PMH as “mental health during the perinatal period” [22]. Whilst the perinatal period is inconsistently defined within literature [22, 23], in this review, we defined the perinatal period as the time from the beginning of pregnancy to one year following the birth or end of the pregnancy [18]. The common mental health disorders which occur during the perinatal period include depression, anxiety and somatic disorders, while psychosis and substance use disorders occur more rarely [22]. This scoping review considered indicators from any setting which aligned with this definition, including both peer-reviewed journal articles and grey literature. After feedback from consultations with WHO and MoNITOR group members, we narrowed our scope and excluded indicators which measure risk factors and social determinants of PMH disorders, and indicators which measured mental health generally but were not specific the perinatal population. The quality of the indicator was not a criterion for inclusion. For the peer reviewed search, we did not limit by language of publication but used translation services for papers in a language other than English. The non-peer-reviewed material was searched in English only due to difficulty with navigation and translation of non-English webpages.

For inclusion, resources must have had:

1. An indicator which specifically measures perinatal mental health.
2. A definition of the indicator.
3. A publication date from 1 January 2000 to 4 October 2024.

The exclusion criteria were that the:

1. Indicator was not specific to perinatal population.
2. Indicator measured a risk factor and social determinant of PMH only.
3. Indicator had not yet been developed.

### Data extraction and analysis

Indicators were extracted individually from each resource. All available indicator data such as: indicator name, definition, numerator, denominator, and data source were compiled (S3 Table). Indicators with similar definitions were grouped together. Where it was determined that the indicators measured the same outcome, we proposed an indicator definition by combining them. (Table 1). The indicators in this core list were then mapped to the relevant level in the PMH ToC. The quality of each indicator was outside the scope of this review and hence was not assessed.

**Table 1:** Core list of indicators mapped against PMH ToC.

Theory of change domain	Indicator short title	Proposed indicator	Indicator from resource
Individual	Postpartum depression	1. Proportion of women with postpartum depression	Postpartum depression as assessed by the PHQ-2 or EPDS [29]
			The number of women who are identified as suffering from post-natal depression, divided by the total number of women [30]
			Percentage of women having a live birth who experienced depressive symptoms after pregnancy [31]
			Percentage of women who have recently given birth who reported experiencing postpartum depression following a live birth [32]
			The estimated number of women with postpartum depressive symptoms [33]
Individual	Perinatal depression and anxiety	2. Proportion of women with depression or anxiety in perinatal period	The estimated number of women with mild-moderate depressive illness and anxiety [34]
			The estimated number of women with severe depressive illness [34]
			Patient-reported depression during antenatal and postnatal care periods [35]
Individual	Postpartum psychosis	3. Proportion of women with postpartum psychosis	Acute psychosis among women during childbirth episode [36–38]
			The estimated number of women with postpartum psychosis [34]
Individual	Adjustment disorders	4. Proportion of women with adjustment disorder and distress in perinatal period	The estimated number of women with adjustment disorders and distress [34]
Individual	Post-Traumatic Stress Disorder	5. Proportion of women with Post-Traumatic Stress Disorder in perinatal period	The estimated number of women with post-traumatic stress disorder [34]
Individual	Mental health conditions	6. Proportion of women with mental health conditions in perinatal period	The estimated number of women with chronic serious mental illness [34]
			Percentage of pregnant women with psychological or psychiatric problems [39, 40]
Service delivery ecosystem	Screening for mental health conditions	7. Proportion of women screened for mental health conditions in perinatal period	Whether screening for mental health risk using a validated screening tool has been conducted during the antenatal period [41]
			Proportion of women who were screened for postpartum depression after a live birth [42]
			Documented evidence that the mother and her family/partner were encouraged to advise their public health nurse about mental health history, changes in mood, emotional state and behaviour that are outside of the mother’s normal pattern. Care plan initiated as appropriate [43]
			Documented evidence that verbal and written information in relation to signs and symptoms of postnatal depression and preventative measures were given to the mother and partner if present. Mother advised to contact public health nurse service if symptoms occur. Care plan initiated as appropriate [43]
			Every pregnant woman is screened during the perinatal period at fixed moments for psychosocial vulnerability [44]
Service delivery ecosystem	Mental health services	8. Proportion of women with access to mental health services during the perinatal period	Whether a woman in the perinatal period had access to mental health services since a disaster [45]
			Access to mental health services during antenatal care [46]
Service delivery ecosystem	Healthcare workers trained to manage psychological problems	9. Proportion of healthcare providers trained to provide mental healthcare in perinatal period	Every health and social care provider has been expertly trained in dealing with psychological and social problems [44]

## Results

**Fig 1:**
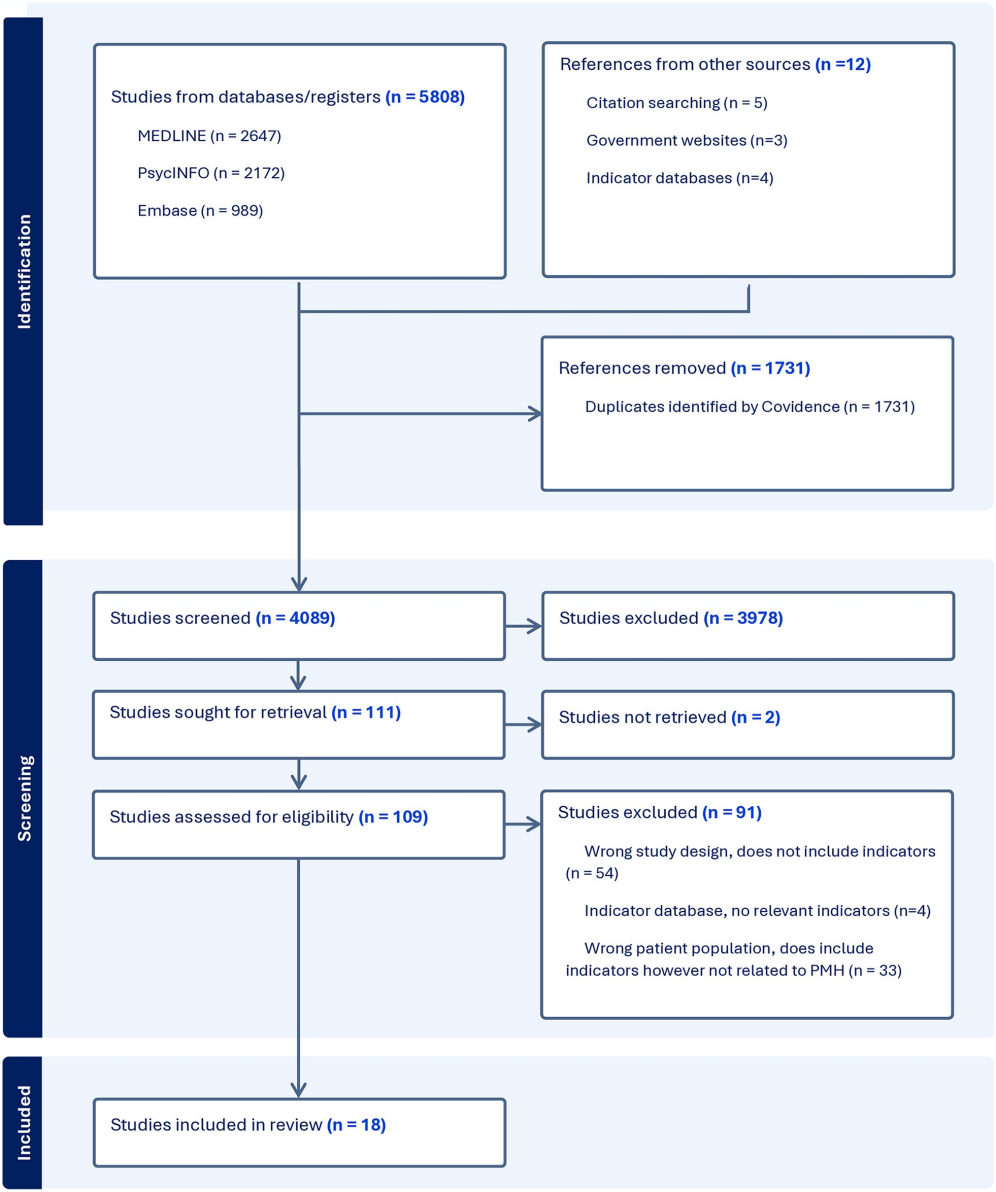
PRISMA flow diagram.

A total of 5808 resources from three databases were retrieved (Fig 1). An additional 12 resources from other sources including government websites and international agency websites were also retrieved. After duplicates were removed 4082 records were screened at the title/abstract stage and 109 at full text. Overall, 2 studies could not be retrieved and 91 were excluded at full text screening, resulting in 18 resources for inclusion.

From the 18 resources which were included, 25 indicators which measured PMH were extracted (S3 Table). Of these, 15 studies came from the database search of peer reviewed material with 17 indicators extracted. The grey literature search of webpages found the additional three resources, with eight indicators extracted. These sources were all from governmental websites (United Kingdom (UK) (n = 6), Australia (n = 1) and the United States of America (U.S.) (n = 1)]. There were no indicators found relating to PMH through searching the webpages of WHO, OECD, MICS or DHS [15, 26–28]. Of the 18 resources, 16 were from HICs (USA, UK, Australia, Ireland, Netherlands and Belgium]) and two were from LMICs (Mexico and Kenya).

The process of compiling indicators resulted in nine core indicators (Table 1). The proposed indicators were mapped to the PMH ToC, with six relating to individual measures of PMH, and three relating to measures of the PMH service delivery ecosystem.

## Discussion

The aim this scoping review was to identify existing perinatal mental health indicators and propose a core set which could be used at a global level. We found 25 indicators for PMH aligned with the PMH ToC, which were condensed to form a core set of nine indicators. This set of indicators can contribute to the development of a monitoring framework which if implemented can measure countries’ progress towards widely accessible, high-quality provision of PMH care services.

We used the PMH ToC as the conceptual framework. Of the five domains of the ToC, we found PMH specific indicators for only two—individual and service delivery ecosystem. Broadly speaking, the included indicators in the individual domain aim to measure the prevalence of PMH disorders, while those identified in the service delivery ecosystem domain aim to measure service provision and quality. We were unable to identify any PMH specific indicators across the other three domains. Hence, many areas recognised as central to improving PMH may be without existing indicators. These, for example, include things such as understanding socio-cultural norms [9], community engagement with PMH promotion [10], the availability of treatment options; counselling and psychotherapy, accessibility of psychotropic medications and availability of inpatient care if required [4, 47], and policies that address PMH [48].

Of the 25 indicators identified for PMH, only two were from papers from an LMIC [Kenya and Mexico], with the rest from high-income countries. Therefore, the majority of the indicators may not be applicable to LMICs. This has also been highlighted in the pilot study by Al-Shammari et al. [35] in Kenya which used the International Consortium of Health Outcomes Measurement developed in the UK, which includes an indicator for postpartum depression. They found significant gaps in the mental health knowledge of their healthcare staff, which required specific training to implement the resource. They also found no women reporting a prior diagnosis of a PMH disorder despite many reporting symptoms [35]. This highlights the importance of global indicators being universally valid.

The WHO recommends that PMH care be integrated into existing maternal and child health services [18]. Whilst this is an important step in improving accessibility of PMH care, additional steps are necessary in advancing towards the goal of universal high-quality PMH care for all perinatal women. A cornerstone of indicator implementation involves the ability of health systems and data collection platforms to monitor progress. Currently, however, there are significant limitations to implementing a core set of PMH indicators as required data is not routinely captured in many countries around the world. A further important step is the alignment of all relevant guidelines to include PMH, this would include updating the WHO recommendations on antenatal care for a positive pregnancy experience to include PMH.

This scoping review has several limitations. The indicator data available was heterogenous and often incomplete. For example, many resources had indicators with incomplete definitions, or excluded the numerator, denominator, or possible data source. The definitions of each indicator also varied across sources, with differing use of terminologies when describing mental health disorders. An inability to populate all indicators and generalisation of some definitions to form a universal set of indicators may limit the feasibility of implementation. The scope of this research also excluded risk factors and social determinants for PMH, which may have addressed gaps in measurement for the other PMH ToC domains. The grey search of existing indicator databases and webpages was in English only, which could have led to an exclusion of indicators from LMICs. However, a lack of PMH indictors in both LMICs and HICs was well-recognised by experts consulted during this review, highlighting the urgent need for further research in this area. We also imposed a time limit on the papers selected (from January 2000). We felt that the last 24 years was an adequate time frame to include contemporary studies, but it is possible that we omitted studies published before this time.

## Conclusion

The identified PMH indicators in this review provide an account of the current landscape of PMH measurement globally and is the first step in the development of a monitoring framework by the WHO to be used by countries to monitor progress and make programme adjustments. Future research would include the development of new indicators which address the gaps in the PMH ToC interpersonal relationship, community, and policy landscape domains. The development and adaptation of indicators in LMICs would also be vital for future implementation in these settings. Important next steps in this work include further consultation with stakeholders to prioritise and operationalise a core set of agreed indicators, and technical assessment of data sources to determine the feasibility of implementing the indicator set.

This review forms part of the foundational work for the development of a global monitoring framework which will monitor progress towards the provision of universal high quality PMH care. This has implications at a country and global level for the measurement and monitoring of PMH, an important component of the Sustainable Development Goals.

## Supporting information

S1 Appendix Scoping review protocol. (DOCX)

S1 Table Preferred Reporting Items for Systematic reviews and Meta-Analyses extension for Scoping Reviews [PRISMA-ScR] checklist. (DOCX)

S2 Table Search strategy. (DOCX)

S3 Table Indicator data from each resource. (DOCX)

## References

[1] McNab S, Fitzgerald L., Stalls S., Bhatti A., Doggett E., Ricca J., et al. A Shared Vision for Improving Perinatal Mental Health in Lowand Middle-Income Countries- A Theory of Change. USAID MOMENTUM, 2021.

[2] Howard LM, Molyneaux E, Dennis C-L, Rochat T, Stein A, Milgrom J. Non-psychotic mental disorders in the perinatal period. The Lancet. 2014;384(9956):1775–88. doi: 10.1016/S0140-6736(14)61276-9 25455248

[3] Jones I, Chandra PS, Dazzan P, Howard LM. Bipolar disorder, affective psychosis, and schizophrenia in pregnancy and the post-partum period. The Lancet. 2014;384(9956):1789–99. doi: 10.1016/S0140-6736(14)61278-2 25455249

[4] Howard L, & Khalifeh H. Perinatal mental health- a review of progress and challenges. World Psychiatry. 2020;19(3):313–27. doi: 10.1002/wps.20769 32931106 PMC7491613

[5] Biaggi A, Conroy S, Pawlby S, Pariante CM. Identifying the women at risk of antenatal anxiety and depression: A systematic review. Journal of affective disorders. 2016;191:62–77. Epub 2015/11/18. doi: 10.1016/j.jad.2015.11.014 . 26650969 PMC4879174

[6] Khalifeh H, Hunt IM, Appleby L, Howard LM. Suicide in perinatal and non-perinatal women in contact with psychiatric services: 15 year findings from a UK national inquiry. The Lancet Psychiatry. 2016;3(3):233–42. doi: 10.1016/S2215-0366(16)00003-1 26781366

[7] Stevenson K, Fellmeth G, Edwards S, Calvert C, Bennett P, Campbell OMR, et al. The global burden of perinatal common mental health disorders and substance use among migrant women: a systematic review and meta-analysis. Lancet Public Health. 2023;8(3):e203–e16. Epub 2023/02/26. doi: 10.1016/S2468-2667(22)00342-5 . 36841561

[8] Gelaye B, Rondon MB, Araya R, Williams MA. Epidemiology of maternal depression, risk factors, and child outcomes in low-income and middle-income countries. Lancet Psychiatry. 2016;3(10):973–82. doi: 10.1016/S2215-0366(16)30284-X . WOS:000396344300025. 27650773 PMC5155709

[9] McNab S DS, Gomez P, Bhatti A, Khadka N, Kenyi E. The Silent Burden: Common Perinatal Mental Disorders in Low- and Middle-Income Countries. Washington, DC.: USAID MOMENTUM., 2021. 10.1186/s12884-022-04589-z PMC9019797 35443652

[10] Manolova G, Waqas A, Chowdhary N, Salisbury TT, Dua T. Integrating perinatal mental healthcare into maternal and perinatal services in low and middle income countries. BMJ. 2023;381:e073343. Epub 2023/05/24. doi: 10.1136/bmj-2022-073343 interests and have the following interests to declare: No interests to declare. 37220917 PMC10203867

[11] Herba CM, Glover V, Ramchandani PG, Rondon MB. Maternal depression and mental health in early childhood: an examination of underlying mechanisms in low-income and middle-income countries. Lancet Psychiatry. 2016;3(10):983–92. doi: 10.1016/S2215-0366(16)30148-1 . WOS:000396344300026. 27650772

[12] Stein A, Pearson RM, Goodman SH, Rapa E, Rahman A, McCallum M, et al. Effects of perinatal mental disorders on the fetus and child. Lancet. 2014;384 North American Edition(9956):1800–19. doi: 10.1016/S0140-6736(14)61277-0 . Language: English. Entry Date: 20150227. Revision Date: 20200708. Publication Type: journal article. 25455250

[13] World Health Organization. Maternal mental health n.d. [cited 2023 1 September 2023]. https://www.who.int/teams/mental-health-and-substance-use/promotion-prevention/maternal-mental-health.

[14] Roddy Mitchell A, Gordon H, Lindquist A, Walker SP, Homer CSE, Middleton A, et al. Prevalence of Perinatal Depression in Low- and Middle-Income Countries: A Systematic Review and Meta-analysis. JAMA Psychiatry. 2023. Epub 2023/03/09. doi: 10.1001/jamapsychiatry.2023.0069 . 36884232 PMC9996459

[15] World Health Organisation. The Global health Observatory, Indicators index 2024 [cited 2023 8 March]. https://www.who.int/data/gho/data/indicators/indicators-index.

[16] World Health Organization. WHO recommendations on maternal and newborn care for a positive postnatal experience. 2022. 35467813

[17] World Health Organisation. Improving early childhood development: WHO guideline. Geneva 2020. 32200595

[18] World Health Organization. Guide for integration of perinatal mental health in maternal and child health services. Geneva: 2022.

[19] The United Nations. The Sustainable Development Goals 2023 [14 May 2023]. www.un.org/sustainabledevelopment/health.

[20] World Health Organization. Health Indicators: conceptual and operational considerations. 2018.

[21] Tricco AC, Lillie E, Zarin W, O’Brien KK, Colquhoun H, Levac D, et al. PRISMA Extension for Scoping Reviews (PRISMA-ScR): Checklist and Explanation. Annals of internal medicine. 2018;169(7):467–73. Epub 2018/09/05. doi: 10.7326/M18-0850 . 30178033

[22] McNab SE, Dryer SL, Fitzgerald L, Gomez P, Bhatti AM, Kenyi E, et al. The silent burden: a landscape analysis of common perinatal mental disorders in low- and middle-income countries. BMC pregnancy and childbirth. 2022;22(1):342. doi: 10.1186/s12884-022-04589-z 35443652 PMC9019797

[23] McNab SE, Fitzgerald L, Stalls S, Bhatti AM, Doggett E, Ricca J, et al. A Shared Vision for Improving Perinatal Mental Health in Low- and Middle-Income Countries: A Theory of Change and Prioritized Implementation Research Questions. USAID, MOMENTUM, 2022.

[24] World Health Organisation. WHO guide for integration of perinatal mental health in maternal and child health services. Geneva: 2022.

[25] Covidence systematic review software. Covidence Melbourne, Australia: Veritas Health Innovation; 2023.

[26] USAID. Demographic and Health Surveys 2024 [cited 2023 8th March]. https://www.dhsprogram.com/data/.

[27] UNICEF. Multiple Indicator Cluster Surveys (MICS) 2024 [cited 2023 8th March]. https://microdata.worldbank.org/index.php/catalog/MICS/?page=1&ps=15&repo=MICS.

[28] Organisation for Economic Co-operation and Development. Leading Indicators 2024 [cited 2023 8th March]. https://www.oecd.org/sdd/leading-indicators/.

[29] Nijagal MA, Wissig S, Stowell C, Olson E, Amer-Wahlin I, Bonsel G, et al. Standardized outcome measures for pregnancy and childbirth, an ICHOM proposal. BMC health services research. 2018;18(1):953. doi: 10.1186/s12913-018-3732-3 30537958 PMC6290550

[30] Troop P, Goldacre M, Mason A, Cleary R. Health Outcome Indicators: Normal Pregnancy and Childbirth. Report of a working group to the Department of Health. Oxford: National Centre for Health Outcomes Development; 1999.

[31] Broussard DL, Sappenfield WB, Fussman C, Kroelinger CD, Grigorescu V. Core state preconception health indicators: a voluntary, multi-state selection process. Maternal and child health journal. 2011;15(2):158–68. doi: 10.1007/s10995-010-0575-x 20225127

[32] Callahan T, Stampfel C, Cornell A, Diop H, Barnes-Josiah D, Kane D, et al. From Theory to Measurement: Recommended State MCH Life Course Indicators. Maternal and child health journal. 2015;19(11):2336–47. doi: 10.1007/s10995-015-1767-1 26122251 PMC4595527

[33] Centers for Disease Control and Prevention Chronic Disease Indicators U.S. Department of Health and Human Services; 2023. https://www.cdc.gov/cdi/.

[34] Office for Health Improvement and Disparities Public health profiles: Department of Health and Social Care; 2023. https://fingertips.phe.org.uk.

[35] Al-Shammari I, Roa L, Yorlets RR, Akerman C, Dekker A, Kelley T, et al. Implementation of an international standardized set of outcome indicators in pregnancy and childbirth in Kenya: Utilizing mobile technology to collect patient-reported outcomes. PLoS One. 2019;14(10):e0222978. Epub 2019/10/17. doi: 10.1371/journal.pone.0222978 Measurement (ICHOM). The feasibility of implementing one of the ICHOM Standard Set was piloted in this study. The financers also do not alter our adherence to PLOS ONE policies on sharing data and materials. 31618249 PMC6795527

[36] Nair M, Kurinczuk JJ, Knight M. Establishing a National Maternal Morbidity Outcome Indicator in England: A Population-Based Study Using Routine Hospital Data. PloS one. 2016;11(4):e0153370. doi: 10.1371/journal.pone.0153370 27054761 PMC4824476

[37] Easter A, Howard LM, Sandall J. Mental health near miss indicators in maternity care: a missed opportunity? A commentary. BJOG: an international journal of obstetrics and gynaecology. 2018;125(6):649–51. doi: 10.1111/1471-0528.14805 28665503 PMC5947649

[38] Roberts CL, Cameron CA, Bell JC, Algert CS, Morris JM. Measuring maternal morbidity in routinely collected health data: development and validation of a maternal morbidity outcome indicator. Medical care. 2008;46(8):786–94. doi: 10.1097/MLR.0b013e318178eae4 18665058

[39] Molenaar JM, Boesveld IC, Kiefte-de Jong JC, Struijs JN. Monitoring the Dutch Solid Start Program: Developing an Indicator Set for Municipalities to Monitor their First Thousand Days-Approach. International journal of integrated care. 2022;22(4):8. doi: 10.5334/ijic.6508 36381589 PMC9615606

[40] Boesveld I, Molenaar J, De Vries E, Struijs J. Indicator set for Local Monitor for a Promising Start: a Delphi Study. Netherlands: National Institute for Public Health and the Environment; 2021.

[41] Australian Institute of Health and Welfare METEOR Metadata Online Registy 2023. https://meteor.aihw.gov.au/.

[42] Korst LM, Gregory KD, Lu MC, Reyes C, Hobel CJ, Chavez GF. A Framework for the Development of maternal quality of care indicators. Maternal and child health journal. 2005;9(3):317–41. doi: 10.1007/s10995-005-0001-y 16160758

[43] Giltenane M, Sheridan A, Kroll T, Frazer K. Identification of quality indicators of public health nursing practice: "modified Delphi" approach. Public health nursing (Boston, Mass). 2022;39(1):214–28. doi: 10.1111/phn.13000 34697828

[44] D’Haenens F, Helsloot K, Lauwaert K, Agache L, de Velde GV, De Frene V, et al. Towards an integrated perinatal care pathway for vulnerable women: The development and validation of quality indicators. Midwifery. 2020;89:102794. doi: 10.1016/j.midw.2020.102794 32668387

[45] Zotti ME, Williams AM, Wako E. Post-disaster health indicators for pregnant and postpartum women and infants. Maternal and child health journal. 2015;19(6):1179–88. doi: 10.1007/s10995-014-1643-4 25476606 PMC11025785

[46] Meneses Navarro S, Serván-Mori E, Heredia-Pi I, Pelcastre B, Nigenda G. Ethnic Disparities in Sexual and Reproductive Health in Mexico After 25 Years of Social Policies. Sexuality Research and Social Policy. 2022;19:1–16. doi: 10.1007/s13178-022-00692-0

[47] Gajaria A, Ravindran AV. Interventions for perinatal depression in low and middle-income countries: A systematic review. Asian Journal of Psychiatry. 2018;37:112–20. doi: 10.1016/j.ajp.2018.08.014 . WOS:000448471300024. 30173015

[48] World Health Organization. Comprehensive Mental Health Action Plan 2013–2030. Geneva: World Health Organization, 2021.

