# Supplementary material for: Maternal mental health matters: Indicators for perinatal mental health—A scoping review
Source: PLoS One. 2025 Jan 27;20(1):e0317998. doi: 10.1371/journal.pone.0317998 (PMC11771939; doi:10.1371/journal.pone.0317998)
Supplement: S1 Appendix — (DOCX) [file pone.0317998.s001.docx]

**S1 Appendix. Scoping review protocol**

**Identifying and Prioritising Indicators for Monitoring Maternal Mental Health**

Team:

Professor Caroline Homer, Dr Elissa Kennedy, Dr Elly Layton & Alexandra Roddy Mitchell

Approved by World Health Organization on 2/5/2023

**Background**

The World Health Organization (WHO) recognises health as “a state of complete physical, mental, and social wellbeing, and not merely the absence of disease or infirmity” ^1p.2^. Mental health, however, remains a neglected part of efforts to improve health globally, and this is particularly so in perinatal women. The perinatal period (from conception to 12 months postpartum) represents a unique time in a woman’s life when there are significant physical and social changes^2^. Mental health disorders are among the most common morbidities experienced by women during this period^3^. Maternal mental health disorders make a significant contribution to maternal morbidity and mortality, and are associated with negative outcomes for neonates, infants, and children^4^.

In women, perinatal mental health disorders are associated with reduced quality of life, substance use disorders, and suicide^5, 6^. They increase the likelihood of women experiencing poverty, physical health complications and intimate partner violence^7^. Further to this, they can impact the mother-infant bond and are associated with poorer neurodevelopmental and emotional outcomes in children^8, 9^. At the societal level, maternal mental health disorders add a significant economic burden^4^. Maternal mental health disorders are highly prevalent, estimated to impact one in four perinatal women^10, 11^. In general prevalence is estimated to be greater in low- and middle-income countries than high income countries^7^.

Healthy women are the cornerstone of healthy societies^12^. Maternal mental health not only impacts the mother but has implications for her child too. Early life experiences are increasingly being understood as fundamental determinants of both mental and physical health later in life^13^. Hence improving maternal mental health not only benefits the woman but has important flow-on effects for her children, family, and the wider community.

Collection of routine key indicators of maternal mental health is essential in formulating and implementing policy, organising service delivery, and shaping systems to best support the needs of the population. Monitoring progress and trends enables efforts to be directed to where they are most effective and continually evaluated. The WHO Special Initiative for Mental Health seeks to ensure universal health coverage in 12 priority countries to 100 million more people^14^. This initiative as well as the WHO Comprehensive Mental Health Action Plan 2013 – 2030 will contribute towards the broader aims of the Sustainable Development Goals. Currently, however, WHO does not have guidelines for monitoring the status of maternal mental health specifically. Recognising maternal mental health as a public health priority that requires focused efforts is essential in realising the Sustainable Development Goals and supporting women’s mental health and wellbeing. Therefore, the purpose of this review is to determine the most effective and practicable indicators of maternal mental health in high, and low- and middle-income settings.

**Methods**

A scoping review based on the framework outlined by Arksey and O’Malley^15^ will be conducted to identify and clarify the concepts underpinning measuring and monitoring maternal mental health and then to synthesise the available evidence of the indicators of maternal mental health. The following research question was formulated: What are the existing indicators for monitoring maternal mental health?

**Search strategy**

The following databases will be systematically searched: MEDLINE, Embase, PsycINFO, using the search method outlined below, developed in consultation with The Alfred Library service.

| **MEDLINE Database search** | |
| --- | --- |
| 1 | Health Status Indicators/ |
| 2 | Quality Indicators, Health Care/ |
| 3 | Indicator*.mp |
| 4 | 1 or 2 or 3 |
| 5 | perinatal care/ or postnatal care/ or peripartum period/ or postpartum period/ or pregnancy trimesters/ or pregnancy/ or gravidity/ or parity/ or parturition/ or pregnancy outcome/ |
| 6 | (Maternal or maternity or Perinatal or Postnatal or Pregnancy or Antenatal or peri-natal or post-natal or peripartum or peri-partum or postpartum or post-partum or ante-natal or perinatal care or postnatal care or pregnancy outcome).mp. |
| 7 | mental disorders/ or anxiety disorders/ or mood disorders/ or "bipolar and related disorders"/ or bipolar disorder/ or depressive disorder/ or neurotic disorders/ or paranoid disorders/ or psychotic disorders/ or schizophrenia/ or stress disorders, post traumatic/ |
| 8 | (mental or psych* or depression or Anxiety or Bipolar or Schizophreni* or post-traumatic stress disorder).mp. |
| 9 | 5 or 6 |
| 10 | 7 or 8 |
| 11 | 4 and 9 and 10 |
| 12 | limit 11 to yr="2000 -Current" |
| **Embase Database search** | |
| 1 | health status indicator/ or clinical indicator/ |
| 2 | (indicator or health status indicator or quality indicator).mp. |
| 3 | perinatal care/ or postnatal care/ or pregnancy/ or pregnancy outcome/ or maternal care/ |
| 4 | (perinatal care or postnatal care or peripartum period or postpartum period or pregnancy trimesters or pregnancy or gravidity or parity or parturition or pregnancy outcome or Maternal or maternity or Perinatal or Postnatal or Pregnancy or Antenatal or peri-natal or post-natal or peripartum or peri-partum or postpartum or post-partum or ante-natal or perinatal care or postnatal care or pregnancy outcome).mp. |
| 5 | mental disease/ or anxiety/ or generalized anxiety disorder/ or mood disorder/ or bipolar disorder/ or antenatal depression/ or postnatal depression/ or depression/ or neurosis/ or psychosis/ or schizophrenia/ or posttraumatic stress disorder/ |
| 6 | (mental disorders or anxiety disorders or mood disorders or "bipolar and related disorders" or bipolar disorder or depressive disorder or neurotic disorders or paranoid disorders or psychotic disorders or schizophrenia or stress disorders, post traumatic or mental or psyc* or depression or anxiety or bipolar or schizophreni* or post-traumatic stress disorder).mp. |
| 7 | 1 or 2 |
| 8 | 3 or 4 |
| 9 | 5 or 6 |
| 10 | 7 and 8 and 9 |
| 11 | limit 10 to yr="2000 -Current" |
| **PsyINFO Database search** | |
| 1 | Health status indicators.mp. |
| 2 | Indicators.mp. |
| 3 | Quality indicators.mp. |
| 4 | 1 or 2 or 3 |
| 5 | perinatal period/ or postnatal period/ or pregnancy/ or pregnancy outcomes/ |
| 6 | (Perinatal care or postnatal care or peripartum period or postpartum period or pregnancy trimesters or gravidity or parity or parturition or maternal or maternity or perinatal or postnatal or pregnancy or antenatal or peri-natal or post-natal or peripartum or peri-partum or postpartum or post-partum or ante-natal or perinatal care or postnatal care or pregnancy outcome).mp. |
| 7 | 5 or 6 |
| 8 | Postpartum Psychosis/ or Postpartum Depression/ or Mental disorders/ or Bipolar Disorder/ or Major Depression/ or Psychiatric Symptoms/ or Persistent Depressive Disorder/ or Anxiety Disorders/ or Neurosis/ or brief psychotic disorder/ or paranoid psychosis/ or schizophrenia/ or posttraumatic stress disorder/ |
| 9 | (mental disorders or anxiety disorders or mood disorders or "bipolar and related disorders" or bipolar disorder or depressive disorder or neurotic disorders or paranoid disorders or psychotic disorders or schizophrenia or stress disorders, post traumatic or mental or psyc* or depression or anxiety or bipolar or schizophreni* or post-traumatic stress disorder or postpartum depression).mp. |
| 10 | 8 or 9 |
| 11 | 4 and 7 and 10 |
| 12 | limit 11 to yr="2000 -Current" |

In addition to studies that are identified in the above search, reference lists of studies meeting the inclusion criteria will also be searched for further studies that are relevant and meet the inclusion criteria.

We will also search grey literature for any existing PMH indicators in use by governments or international agencies.

**Inclusion criteria**

Studies will be included if they meet the following criteria:

1. Report on maternal mental health
2. Published in a peer-review journal between January 2000 and April 2023
3. Provide evidence for an indicator or measure of mental health

**Exclusion criteria**

Studies will be excluded if:

1. The population includes people other than mothers/childbearing women
2. Studies where mental health is not the primary outcome measured or indicated
3. Opinion pieces and case studies will be excluded

**Study selection process and data charting**

All studies identified in the systematic search presented above will be uploaded into Covidence systematic review software^16^. Duplicate studies will be removed. First, titles and abstracts will be screened independently by two reviewers (ARM & EL), with any discrepancies decided by a third reviewer (CH or EK). Full texts of studies will then be assessed for eligibility using the same method. A data charting template will be developed, and data charting will be undertaken independently by two reviewers (ARM & EL). Frequent discussions between reviewers will allow for amendments to be made to the data charting from in an iterative process. The purpose of this is to allow mapping of concepts to be guided by the evidence identified in the literature.

The following data will be extracted: year, country, country income level, study type, time period conducted (perinatal, antenatal or postnatal), mental health disorder type, indicator/measure, outcome of indicator (if applicable).

The review will not include assessment of study quality or risk of bias of studies meeting inclusion criteria.

The review will be conducted in accordance with Preferred Reporting Items for Systematic Reviews and Meta-analysis-Scoping Review guidelines^17^.

**Prepare final report**

The indicators identified in this review will be mapped and the Theory of Change^18^ framework will be used to structure the report. Preliminary findings will be presented at a meeting of the WHO Mother and Newborn Information Tracking Outcomes and Results (MoNITOR) group for consultation and refinement.

**References**

1. World Health Organization. Comprehensive Mental Health Action Plan 2013–2030. Geneva: World Health Organization; 2021.

2. Jones I, Chandra PS, Dazzan P, Howard LM. Bipolar disorder, affective psychosis, and schizophrenia in pregnancy and the post-partum period. The Lancet. 2014;384(9956):1789-99.

3. Howard LM, Molyneaux E, Dennis C-L, Rochat T, Stein A, Milgrom J. Non-psychotic mental disorders in the perinatal period. The Lancet. 2014;384(9956):1775-88.

4. Howard LM KH. Perinatal mental health- a review of progress and challenges. World Psychiatry. 2020;19(3):313-27.

5. Khalifeh H, Hunt IM, Appleby L, Howard LM. Suicide in perinatal and non-perinatal women in contact with psychiatric services: 15 year findings from a UK national inquiry. The Lancet Psychiatry. 2016;3(3):233-42.

6. Stevenson K, Fellmeth G, Edwards S, Calvert C, Bennett P, Campbell OMR, et al. The global burden of perinatal common mental health disorders and substance use among migrant women: a systematic review and meta-analysis. Lancet Public Health. 2023;8(3):e203-e16.

7. Gelaye B, Rondon MB, Araya R, Williams MA. Epidemiology of maternal depression, risk factors, and child outcomes in low-income and middle-income countries. Lancet Psychiatry. 2016;3(10):973-82.

8. Herba CM, Glover V, Ramchandani PG, Rondon MB. Maternal depression and mental health in early childhood: an examination of underlying mechanisms in low-income and middle-income countries. Lancet Psychiatry. 2016;3(10):983-92.

9. Stein A, Pearson RM, Goodman SH, Rapa E, Rahman A, McCallum M, et al. Effects of perinatal mental disorders on the fetus and child. Lancet. 2014;384 North American Edition(9956):1800-19.

10. Fisher J, Cabral de Mello M, Patel V, Rahman A, Tran T, Holton S, et al. Prevalence and determinants of common perinatal mental disorders in women in low- and lower-middle-income countries: a systematic review. Bull World Health Organ. 2012;90(2):139G-49G.

11. Roddy Mitchell A, Gordon H, Lindquist A, Walker SP, Homer CSE, Middleton A, et al. Prevalence of Perinatal Depression in Low- and Middle-Income Countries: A Systematic Review and Meta-analysis. JAMA Psychiatry. 2023.

12. Marquez PV. World Bank Blogs. 2017. [cited 2023]. Available from: https://blogs

.worldbank.org/health/healthy-women-are-cornerstone-healthy-societies.

13. Patel V, Saxena S, Lund C, Thornicroft G, Baingana F, Bolton P, et al. The Lancet Commission on global mental health and sustainable development. The Lancet. 2018;392(10157):1553-98.

14. World Health Organization. The WHO Special Initiative for Mental Health (2019-2023)- Universal Health Coverage for Mental Health. 2019.

15. Arksey H, O'Malley L. Scoping studies: towards a methodological framework. International Journal of Social Research Methodology. 2005;8(1):19-32.

16. Covidence systematic review software. Covidence Melbourne, Australia: Veritas Health Innovation; 2023.

17. Tricco AC, Lillie E, Zarin W, O'Brien KK, Colquhoun H, Levac D, et al. PRISMA Extension for Scoping Reviews (PRISMA-ScR): Checklist and Explanation. Ann Intern Med. 2018;169(7):467-73.

18. World Health Organization. Theory of Change. 2020.
