# Supplementary material for: Maternal mental health matters: Indicators for perinatal mental health—A scoping review
Source: PLoS One. 2025 Jan 27;20(1):e0317998. doi: 10.1371/journal.pone.0317998 (PMC11771939; doi:10.1371/journal.pone.0317998)
Supplement: S2 Table — (DOCX) [file pone.0317998.s003.docx]

### **S2 Table. Search strategy**

| **MEDLINE Database search** | |
| --- | --- |
| 1 | Health Status Indicators/ |
| 2 | Quality Indicators, Health Care/ |
| 3 | Indicator*.mp |
| 4 | 1 or 2 or 3 |
| 5 | perinatal care/ or postnatal care/ or peripartum period/ or postpartum period/ or pregnancy trimesters/ or pregnancy/ or gravidity/ or parity/ or parturition/ or pregnancy outcome/ |
| 6 | (Maternal or maternity or Perinatal or Postnatal or Pregnancy or Antenatal or peri-natal or post-natal or peripartum or peri-partum or postpartum or post-partum or ante-natal or perinatal care or postnatal care or pregnancy outcome).mp. |
| 7 | mental disorders/ or anxiety disorders/ or mood disorders/ or "bipolar and related disorders"/ or bipolar disorder/ or depressive disorder/ or neurotic disorders/ or paranoid disorders/ or psychotic disorders/ or schizophrenia/ or stress disorders, post traumatic/ |
| 8 | (mental or psych* or depression or Anxiety or Bipolar or Schizophreni* or post-traumatic stress disorder).mp. |
| 9 | 5 or 6 |
| 10 | 7 or 8 |
| 11 | 4 and 9 and 10 |
| 12 | limit 11 to yr="2000 -Current" |
| **Embase Database search** | |
| 1 | health status indicator/ or clinical indicator/ |
| 2 | (indicator or health status indicator or quality indicator).mp. |
| 3 | perinatal care/ or postnatal care/ or pregnancy/ or pregnancy outcome/ or maternal care/ |
| 4 | (perinatal care or postnatal care or peripartum period or postpartum period or pregnancy trimesters or pregnancy or gravidity or parity or parturition or pregnancy outcome or Maternal or maternity or Perinatal or Postnatal or Pregnancy or Antenatal or peri-natal or post-natal or peripartum or peri-partum or postpartum or post-partum or ante-natal or perinatal care or postnatal care or pregnancy outcome).mp. |
| 5 | mental disease/ or anxiety/ or generalized anxiety disorder/ or mood disorder/ or bipolar disorder/ or antenatal depression/ or postnatal depression/ or depression/ or neurosis/ or psychosis/ or schizophrenia/ or posttraumatic stress disorder/ |
| 6 | (mental disorders or anxiety disorders or mood disorders or "bipolar and related disorders" or bipolar disorder or depressive disorder or neurotic disorders or paranoid disorders or psychotic disorders or schizophrenia or stress disorders, post traumatic or mental or psyc* or depression or anxiety or bipolar or schizophreni* or post-traumatic stress disorder).mp. |
| 7 | 1 or 2 |
| 8 | 3 or 4 |
| 9 | 5 or 6 |
| 10 | 7 and 8 and 9 |
| 11 | limit 10 to yr="2000 -Current" |
| **PsyINFO Database search** | |
| 1 | Health status indicators.mp. |
| 2 | Indicators.mp. |
| 3 | Quality indicators.mp. |
| 4 | 1 or 2 or 3 |
| 5 | perinatal period/ or postnatal period/ or pregnancy/ or pregnancy outcomes/ |
| 6 | (Perinatal care or postnatal care or peripartum period or postpartum period or pregnancy trimesters or gravidity or parity or parturition or maternal or maternity or perinatal or postnatal or pregnancy or antenatal or peri-natal or post-natal or peripartum or peri-partum or postpartum or post-partum or ante-natal or perinatal care or postnatal care or pregnancy outcome).mp. |
| 7 | 5 or 6 |
| 8 | Postpartum Psychosis/ or Postpartum Depression/ or Mental disorders/ or Bipolar Disorder/ or Major Depression/ or Psychiatric Symptoms/ or Persistent Depressive Disorder/ or Anxiety Disorders/ or Neurosis/ or brief psychotic disorder/ or paranoid psychosis/ or schizophrenia/ or posttraumatic stress disorder/ |
| 9 | (mental disorders or anxiety disorders or mood disorders or "bipolar and related disorders" or bipolar disorder or depressive disorder or neurotic disorders or paranoid disorders or psychotic disorders or schizophrenia or stress disorders, post traumatic or mental or psyc* or depression or anxiety or bipolar or schizophreni* or post-traumatic stress disorder or postpartum depression).mp. |
| 10 | 8 or 9 |
| 11 | 4 and 7 and 10 |
| 12 | limit 11 to yr="2000 -Current" |

### Note: All searches were conducted in Ovid. Subject headings are designated by a / symbol. Keyword searching is designated by an .mp.
