# Supplementary material for: Maternal mental health matters: Indicators for perinatal mental health—A scoping review
Source: PLoS One. 2025 Jan 27;20(1):e0317998. doi: 10.1371/journal.pone.0317998 (PMC11771939; doi:10.1371/journal.pone.0317998)
Supplement: S3 Table — (DOCX) [file pone.0317998.s004.docx]

**S3 Table. Indicator data extracted from each resource**

| **Indicator name** | **Definition** | **Numerator** | **Denominator** | **Data source** | **Country** | **Reference** |
| --- | --- | --- | --- | --- | --- | --- |
| Mild-moderate depressive illness and anxiety in perinatal period | The estimated number of women with mild-moderate depressive illness and anxiety | The national prevalence estimate | Total number of maternities (including stillbirth deliveries) | Births, deaths and marriages, Office of National Statistics | UK | (34) |
| Severe depressive illness in perinatal period | The estimated number of women with severe depressive illness | The national prevalence estimate | Total number of maternities (including stillbirth deliveries) | Births, deaths and marriages, Office of National Statistics | UK | (34) |
| Postpartum psychosis | The estimated number of women with postpartum psychosis | The national prevalence estimate | Total number of maternities (including stillbirth deliveries) | Births, deaths and marriages, Office of National Statistics | UK | (34) |
| Chronic severe mental illness in perinatal period | The estimated number of women with chronic serious mental illness | The national prevalence estimate | Total number of maternities (including stillbirth deliveries) | Births, deaths and marriages, Office of National Statistics | UK | (34) |
| PTSD in perinatal period | The estimated number of women with post-traumatic stress disorder | The national prevalence estimate | Total number of maternities (including stillbirth deliveries) | Births, deaths and marriages, Office of National Statistics | UK | (34) |
| Adjustment disorders and distress in perinatal period | The estimated number of women with adjustment disorders and distress | The national prevalence estimate | Total number of maternities (including stillbirth deliveries) | Births, deaths and marriages, Office of National Statistics | UK | (34) |
| Antenatal mental health risk screening status | Whether screening for mental health risk using a validated screening tool has been conducted during the antenatal period | N/A | N/A | N/A | Australia | (41) |
| Postpartum depressive symptoms | The estimated number of women with postpartum depressive symptoms | Respondents who reported that they felt down, depressed, or hopeless, often or always after their most recent live birth | Respondents who reported that they felt down, depressed, or hopeless never, rarely, sometimes, often, or always after delivery of their most recent live birth (excluding unknowns and refusals) | Pregnancy risk assessment monitoring system (PRAMS) a state-based survey of the CDC and health department | U.S. | (33) |
| Psychosocial vulnerability screening | Every pregnant woman is screened during the perinatal period at fixed moments for psychosocial vulnerability. | The percentage of mothers screened for psychosocial vulnerability at fixed times during the perinatal period. | The total number of mothers. | Validated screening tool | Belgium | (44) |
| Healthcare worker training in psychological problems | Every health and social care provider has been expertly trained in dealing with psychological and social problems. | N/A: at organisational level | N/A: at organisational level | N/A | Belgium | (44) |
| Postpartum depression | Assessed by the PHQ-2 or EPDS | N/A | N/A | Patient reported data – PHQ-2 or EPDS | UK | (29) |
| Incidence of post-natal depression | For a population of women delivering within a given time period: the number of  women who are identified as suffering from post-natal depression, divided by the total number of women  operationally defined with reference to the EPDS | The number of women identified as suffering from post-natal depression | Total number of women delivering within a given time period | Patient reported data - EPDS | UK | (30) |
| Postpartum depression | Percentage of women having a live birth who experienced depressive symptoms after pregnancy | N/A | N/A | Pregnancy risk assessment monitoring system (PRAMS) a state-based survey of the CDC and health department | U.S. | (31) |
| Postpartum depression | Percentage of women who have recently given birth who reporting experiencing postpartum depression following a live birth | N/A | N/A | Pregnancy risk assessment monitoring system (PRAMS) a state-based survey of the CDC and health department | U.S. | (32) |
| Depression during pre or post-natal period | Patient-reported depression during antenatal and postnatal care periods | N/A | N/A | Patient reported – survey | Kenya | (35) |
| Acute psychosis | Acute psychosis among women during childbirth episode | Number of women with acute psychosis | Per 10,000 women giving birth | Hospital data | UK | (36-38) |
| Percentage of pregnant women with psychological or psychiatric problems | Percentage of pregnant women with psychological or psychiatric problems | Number of women with healthcare expenditures for mental healthcare services and/or medication related to psychological or psychiatric problems in year of childbirth | Number of pregnant women in year of childbirth | Central bureau of statistics-microdata through DIAPER* - Dutch national dataset | Netherlands | (39, 40) |
| Postpartum depression screening | Proportion of women who were screened for postpartum depression after a live birth | N/A | N/A | Claims based data, healthcare effectiveness data and information set (HEDIS) | U.S. | (42) |
| Encouraged to report changes in mood | Documented evidence that the mother and her family/partner were encouraged to advise their public health nurse about mental health history, changes in mood, emotional state and behaviour that are outside of the mother’s normal pattern. Care plan initiated as appropriate | N/A | N/A | N/A | Ireland | (43) |
| Postnatal depression | Documented evidence that verbal and written information in relation to signs and symptoms of postnatal depression and preventative measures were given to the mother and partner if present. Mother advised to contact public health nurse service if symptoms occur. Care plan initiated as appropriate. | N/A | N/A | N/A | Ireland | (43) |
| Access to mental health services since disaster | Whether a woman in the perinatal period had access to mental health services since a disaster | N/A | N/A | Patient reported data - survey | U.S. | (45) |
| Access to mental health services during antenatal care | N/A | N/A | N/A | National health and nutrition examination survey | Mexico | (46) |
