# Supplementary material for: Maternal mental health matters: Indicators for perinatal mental health—A scoping review
Source: PLoS One. 2025 Jan 27;20(1):e0317998. doi: 10.1371/journal.pone.0317998 (PMC11771939; doi:10.1371/journal.pone.0317998)
Supplement: Attachment — Submitted filename: Rebuttal letter .docx [file pone.0317998.s005.docx]

Dr Elly Layton

Burnet Institute

Melbourne, Australia

15/10/2024

Editorial Office

PLOS ONE

Dear Editorial Office,

Thank you for the feedback regarding a previous submission of our manuscript *“Maternal Mental Health Matters: A scoping review of indicators for perinatal mental health”* (submission PONE-D-24-41021) that the review may be out of date.

Following this, we have repeated the search of the literature and updated the manuscript accordingly. We are confident our new submission provides an up-to-date overview of current indicators for maternal mental health.

Sincerely,

Dr Elly Layton

Burnet Institute

Melbourne, Australia

Elly.layton@burnet.edu.au
