# Supplementary material for: Maternal mental health matters: Indicators for perinatal mental health—A scoping review
Source: PLoS One. 2025 Jan 27;20(1):e0317998. doi: 10.1371/journal.pone.0317998 (PMC11771939; doi:10.1371/journal.pone.0317998)
Supplement: Attachment — Submitted filename: Response to reviewers.docx [file pone.0317998.s006.docx]

Dr Elly Layton

Burnet Institute

Melbourne, Australia

17/12/2024

Editorial Office

PLOS ONE

Dear Editorial Office,

Thank you for the feedback regarding: ***Maternal Mental Health Matters: Indicators for Perinatal Mental Health - A Scoping Review***

Our response to each point from the reviewers can be found below. Please note we have also changed the title following feedback from reviewers.

Our financial disclosure statement should be expanded to state: ‘EL, CSEH and EK received a funding from the **World Health Organisation** (WHO) in Geneva to undertake this review. They worked in collaboration with WHO in relation to the study design, data collection and analysis, decision to publish and preparation of the manuscript. The WHO colleagues are co-authors of the paper.

The authors have declared that no competing interests exist.

Sincerely,

Dr Elly Layton

Burnet Institute

Melbourne, Australia

Elly.layton@burnet.edu.au

1. When submitting your revision, we need you to address these additional requirements.

Please ensure that your manuscript meets PLOS ONE's style requirements, including those for file naming. The PLOS ONE style templates can be found at
[https://journals.plos.org/plosone/s/file?id=wjVg/PLOSOne_formatting_sample_main_body.pdf](https://track.editorialmanager.com/CL0/https:%2F%2Fjournals.plos.org%2Fplosone%2Fs%2Ffile%3Fid=wjVg%2FPLOSOne_formatting_sample_main_body.pdf/1/010f01934f9098bd-6c04a901-b49d-4be4-ae63-610e2f87aaff-000000/aaCpcvSYV50oxuh92xHMdIZIVN-s9N59P3uSR2ax1iY=186) and
[https://journals.plos.org/plosone/s/file?id=ba62/PLOSOne_formatting_sample_title_authors_affiliations.pdf](https://track.editorialmanager.com/CL0/https:%2F%2Fjournals.plos.org%2Fplosone%2Fs%2Ffile%3Fid=ba62%2FPLOSOne_formatting_sample_title_authors_affiliations.pdf/1/010f01934f9098bd-6c04a901-b49d-4be4-ae63-610e2f87aaff-000000/q3C9WYMLx3A0hEBKhMl5YaPuIdBwRUzBGfI_mZgesDY=186)

We have modified file names to meet PLOS One’s style requirements.

2. Please expand the acronym WHO (as indicated in your financial disclosure) so that it states the name of your funders in full.
This information should be included in your cover letter; we will change the online submission form on your behalf.

The acronym has been expanded in our cover letter and should read ‘World Health Organization’. Thank you for modifying this information in the online submission.

3. Thank you for stating the following in your Competing Interests section: [No].

Please complete your Competing Interests on the online submission form to state any Competing Interests. If you have no competing interests, please state "The authors have declared that no competing interests exist.", as detailed online in our guide for authors at [http://journals.plos.org/plosone/s/submit-now](http://track.editorialmanager.com/CL0/http:%2F%2Fjournals.plos.org%2Fplosone%2Fs%2Fsubmit-now/1/010f01934f9098bd-6c04a901-b49d-4be4-ae63-610e2f87aaff-000000/kz42fqP4ycn7j-Ifl-viKy5lpnSY6xMFhhkINTYOa_Y=186)

This information should be included in your cover letter; we will change the online submission form on your behalf.

Our cover letter now contains this information. Thank you for changing this information in the online submission.

4. We note that your Data Availability Statement is currently as follows: [All relevant data are within the manuscript and its Supporting Information files.]

Please confirm at this time whether or not your submission contains all raw data required to replicate the results of your study. Authors must share the “minimal data set” for their submission. PLOS defines the minimal data set to consist of the data required to replicate all study findings reported in the article, as well as related metadata and methods ([https://journals.plos.org/plosone/s/data-availability#loc-minimal-data-set-definition](https://track.editorialmanager.com/CL0/https:%2F%2Fjournals.plos.org%2Fplosone%2Fs%2Fdata-availability%23loc-minimal-data-set-definition/1/010f01934f9098bd-6c04a901-b49d-4be4-ae63-610e2f87aaff-000000/YN6-G_HesXXHA1had44qW8tZWIFM0TLgblnqj-zEIm4=186)).

For example, authors should submit the following data:

- The values behind the means, standard deviations and other measures reported;
- The values used to build graphs;
- The points extracted from images for analysis.

Authors do not need to submit their entire data set if only a portion of the data was used in the reported study.

If your submission does not contain these data, please either upload them as Supporting Information files or deposit them to a stable, public repository and provide us with the relevant URLs, DOIs, or accession numbers. For a list of recommended repositories, please see [https://journals.plos.org/plosone/s/recommended-repositories](https://track.editorialmanager.com/CL0/https:%2F%2Fjournals.plos.org%2Fplosone%2Fs%2Frecommended-repositories/1/010f01934f9098bd-6c04a901-b49d-4be4-ae63-610e2f87aaff-000000/p2VIFtcN5QshazIlZTsoAMWbFSB_PzBcPfxKHfgKQ5E=186).

If there are ethical or legal restrictions on sharing a de-identified data set, please explain them in detail (e.g., data contain potentially sensitive information, data are owned by a third-party organization, etc.) and who has imposed them (e.g., an ethics committee). Please also provide contact information for a data access committee, ethics committee, or other institutional body to which data requests may be sent. If data are owned by a third party, please indicate how others may request data access.

We can confirm that the submission contains all raw data to replicate the results of the study.

5. We notice that your supplementary files are included in the manuscript file. Please remove them and upload them with the file type 'Supporting Information'. Please ensure that each Supporting Information file has a legend listed in the manuscript after the references list.

Supplementary files have been removed and uploaded separately as per the PLOS One style template.

[Note: HTML markup is below. Please do not edit.]

Reviewers' comments:

Reviewer's Responses to Questions

**Comments to the Author**

1. Is the manuscript technically sound, and do the data support the conclusions?

The manuscript must describe a technically sound piece of scientific research with data that supports the conclusions. Experiments must have been conducted rigorously, with appropriate controls, replication, and sample sizes. The conclusions must be drawn appropriately based on the data presented.

Reviewer #1: Partly

Reviewer #2: Yes

2. Has the statistical analysis been performed appropriately and rigorously?

Reviewer #1: N/A

Reviewer #2: N/A

3. Have the authors made all data underlying the findings in their manuscript fully available?

The [PLOS Data policy](http://track.editorialmanager.com/CL0/http:%2F%2Fwww.plosone.org%2Fstatic%2Fpolicies.action%23sharing/1/010f01934f9098bd-6c04a901-b49d-4be4-ae63-610e2f87aaff-000000/nh26JPvelEyCiUabTmaTKd8-CBq2PTHdKLq49d8cn2s=186) requires authors to make all data underlying the findings described in their manuscript fully available without restriction, with rare exception (please refer to the Data Availability Statement in the manuscript PDF file). The data should be provided as part of the manuscript or its supporting information, or deposited to a public repository. For example, in addition to summary statistics, the data points behind means, medians and variance measures should be available. If there are restrictions on publicly sharing data—e.g. participant privacy or use of data from a third party—those must be specified.

Reviewer #1: Yes

Reviewer #2: Yes

4. Is the manuscript presented in an intelligible fashion and written in standard English?

PLOS ONE does not copyedit accepted manuscripts, so the language in submitted articles must be clear, correct, and unambiguous. Any typographical or grammatical errors should be corrected at revision, so please note any specific errors here.

Reviewer #1: Yes

Reviewer #2: Yes

5. Review Comments to the Author

Please use the space provided to explain your answers to the questions above. You may also include additional comments for the author, including concerns about dual publication, research ethics, or publication ethics. (Please upload your review as an attachment if it exceeds 20,000 characters)

Reviewer #1: Very pertinent study, with good theoretical foundation.
The title of an SR must always end with: A Scoping Review and not in the way it is presented.

Thank you for your feedback. We have changed the title accordingly to now read: “Maternal Mental Health Matters: Indicators for Perinatal Mental Health - ~~:~~ A Scoping Review”

The abstract must contain a set of information that is not clear in the study. They mention that they carry out an SR but do not follow the principles of their research protocol and the recommendation (Preferred Reporting Items for Systematic reviews and Meta-Analyses extension for Scoping Reviews (PRISMA-ScR) Checklist).

We have edited the Abstract to provide the key information more clearly. We have followed the principles of a scoping review. This is detailed in the PRIMSA-ScR checklist which is now found in S1.

Do not put acronyms on abstract. There are acronyms that are not spelled out, for example CDC.

This has been addressed. We have ensured all acronyms have been spelled out.

It is recommended that the SR protocol is registered and included in the Open Science Framework (OSF), for example, before publishing the report.

The scoping review is not registered, however our protocol has now been added as a supplementary file (S1 Appendix).

They should not have chronological lines to map out all the evidence on the topic under study.

We are not clear as to what this comment refers to, so we have been unable to respond. We are unclear of the meaning of this comment, if further clarification could be provided, we would be happy to address it.

It would have been easier if they had included the gray literature they included in the research in the diagram.

We have also now included grey literature in the PRISMA flow diagram (Fig 1) and included an exclusion criterion in the body of the manuscript. Rationale for choice of grey literature has also been included.

It is confusing for anyone who reads how you did the research and how you arrived at the results. When extracting the results, I would like to have seen the PRISMA flow diagram explained. From what they seem, it is not clear how they proceeded with data extraction and analysis. Dot provides numbers of evidence sources selected, assessed for eligibility and included in the review, with reasons for exclusions at each stage, using the flow diagram.

Thank you for this feedback. We have now explained the PRISMA flow diagram in the results section and included it as Figure 1 (opposed to as a supplementary file). Results section (line 150) now reads:

“A total of 5808 resources from three databases were retrieved (Figure 1). An additional 12 resources from other sources including government websites and international agency websites were also retrieved. After duplicates were removed 4082 records were screened at the title/abstract stage and 109 at full text. Overall, 2 studies could not be retrieved and 91 were excluded at full text screening, resulting in 18 resources for inclusion.”

Removal of results is unclear. Very confusing. Do not state the process for selecting sources of evidence (i.e., screening and eligibility) included in the scoping review. In the "Summary of indicators mapped against PMH ToC" and the "Supplementary file 3.

We have clarified this and included more about what we did in the Methods and the results of this are now in the Findings. We hope that makes it clearer.

Indicator data extracted from each resource", there are incorrect references to all resources. in the text it presents one type of references and in the table another. must be uniform in accordance with the magazine's requests. Korst et al (US) no references nº (Table1).

References have been updated to be consistent throughout the text and tables.

It would be interesting to provide a general interpretation of the results in relation to the review questions and objectives, as well as possible implications.

We have revised the Discussion to address this comment. The possible implications have also been clarified in the discussion.

Reviewer #2: Mertodologia:

A search was carried out (Medline, Embase PsycInfo ). Why didn't you search Pubmed, the largest health database?

We did not search the PubMed interface as we searched the Medline database directly through the Ovid interface.

The term perinatal period | No results found as a DeCS/MeSH term, Health Sciences Descriptors

S2 Table includes all search terms used. The term perinatal was used however pregnancy, postnatal, peripartum, etc were also used. This was done with the aim of capturing all relevant resources even if some were specific to pregnancy or postpartum, but therefore still meet the definition of the perinatal period.

The terms mental health and indicator correspond to several DeCS/MeSH and Health Sciences Descriptors, so it's essential to include the ID to identify which one was used in the search.
Supplementary file one does not mention which Boolean phrases were used in the search, so we could not replicate the search.

We have added a note to the supplementary table which now describes which search terms were MeSH terms/keywords and the search should therefore be reproducible.

We also searched Governmental websites from English-speaking countries; it would be essential to mention the phrase in natural language as they did that search.

We have now included more information on how we searched governmental websites in the methods. Methods section (line 112) now reads: “For each of these resources, available indicator dashboards were systemically reviewed for relevant indicators and the keyword ‘perinatal mental health’ was searched.”

In the Prisma flowchart, it would be necessary to mention whether or not there were findings in this way and through direct contact with researchers (grey literature).

The methods have also been updated to expand the search strategy of the grey resources. These have also been included in the PRISMA (Fig 1). We did not find any extra resources through contact with researchers, which we have now stated in the methods.

The description of the results should include the overall number of articles found or the PRISMA flowchart to make it easier to understand.

Thank you for this feedback. We have now explained the PRISMA flow diagram in the results section and included it as Figure 1 (opposed to as a supplementary file). Results section (line 150) now reads:

“A total of 5808 resources from three databases were retrieved (Figure 1). An additional 12 resources from other sources including government websites and international agency websites were also retrieved. After duplicates were removed 4082 records were screened at the title/abstract stage and 109 at full text. Overall, 2 studies could not be retrieved and 91 were excluded at full text screening, resulting in 18 resources for inclusion.”

Very confusing discussion:
They present the limitations of the review in the discussion and should emphasise them at the end of the article.
Including a short conclusion withgs and implications for practice would be essential the main findin.

We have worked on the Discussion to improve the flow. We have clarified the implications of this work in the discussion.

6. PLOS authors have the option to publish the peer review history of their article ([what does this mean?](https://track.editorialmanager.com/CL0/https:%2F%2Fjournals.plos.org%2Fplosone%2Fs%2Feditorial-and-peer-review-process%23loc-peer-review-history/1/010f01934f9098bd-6c04a901-b49d-4be4-ae63-610e2f87aaff-000000/xT9kTFF4ZUD2IkoqCEv2r3MM0Y9Sz9pxr1foIDCwTek=186)). If published, this will include your full peer review and any attached files.


If you choose “no”, your identity will remain anonymous but your review may still be made public.


**Do you want your identity to be public for this peer review?** For information about this choice, including consent withdrawal, please see our [Privacy Policy](https://track.editorialmanager.com/CL0/https:%2F%2Fwww.plos.org%2Fprivacy-policy/1/010f01934f9098bd-6c04a901-b49d-4be4-ae63-610e2f87aaff-000000/3ktBK5cjlNS00ZX-EjwwIUoEGWuRwfBp52qbo7pYQvk=186).

Reviewer #1: **Yes:** Sara Palma

Reviewer #2: **Yes:** Delfina Ana Pereira Ramos Teixeira

[NOTE: If reviewer comments were submitted as an attachment file, they will be attached to this email and accessible via the submission site. Please log into your account, locate the manuscript record, and check for the action link "View Attachments". If this link does not appear, there are no attachment files.]

While revising your submission, please upload your figure files to the Preflight Analysis and Conversion Engine (PACE) digital diagnostic tool, [https://pacev2.apexcovantage.com/](https://track.editorialmanager.com/CL0/https:%2F%2Fpacev2.apexcovantage.com%2F/1/010f01934f9098bd-6c04a901-b49d-4be4-ae63-610e2f87aaff-000000/K3ADxLssY3xAi0sUue_bMwS_uKPOzxRs7n1TI_WTT4Y=186). PACE helps ensure that figures meet PLOS requirements. To use PACE, you must first register as a user. Registration is free. Then, login and navigate to the UPLOAD tab, where you will find detailed instructions on how to use the tool. If you encounter any issues or have any questions when using PACE, please email PLOS at [figures@plos.org](mailto:figures@plos.org). Please note that Supporting Information files do not need this step.
