# Supplementary material for: Maternal mental health matters: Indicators for perinatal mental health—A scoping review
Source: PLoS One. 2025 Jan 27;20(1):e0317998. doi: 10.1371/journal.pone.0317998 (PMC11771939; doi:10.1371/journal.pone.0317998)
Supplement: Attachment — Submitted filename: Response to reviewers R2.docx [file pone.0317998.s007.docx]

**PONE-D-24-46366R1**

**Maternal Mental Health Matters: Indicators for Perinatal Mental Health - A Scoping Review**

**PLOS ONE**

Thank you for the feedback regarding: Maternal Mental Health Matters: Indicators for Perinatal Mental Health - A Scoping Review.

Our response to each point from the reviewers can be found below

**Reviewer #1: Thank you very much for your concern in making the necessary rectifications.
Regarding the time limits for research. The authors selected the publication date limit from 1 January 2000 to 4 October 2024. I question why they selected this period, since ScR should not have a period for research?**

We wanted to include recent research and felt that the year 2000 was as far back as we wanted to go. We have added that justification in the Methods and also a line under limitations.

**However, i suggested, what they show in "data extraction and analysis", lines 135-140, must be presented in the results and what they show in lines 143-147 in "data extraction and analysis".**

Lines 135-140 are the section under Data extraction and analysis. This is clearly the process which was undertaken and not the findings so we would prefer to keep it in the Methods as is commonly presented this way.

Lines 143-147 do present the findings – the results of the search process.

**Lastly, in the discussion of the results, there is no comparison of authors. I think it would enrich the results.**

We are not really sure what is meant here. We have never presented a comparison of authors before in a scoping review. The authors are all listed in the supplementary files.

**Reviewer #2: The authors present the methodology using the PRIMA flowchart and complement it with annexes, making the results much easier to understand.
The way they present the results of the articles also makes them easier to read and understand, as does the discussion.
However, they could separate the conclusion or a summary and the limitations of discussing the results.**

There is a paragraph on the limitations already within the Discussion.

We have made a heading for the Conclusion as suggested.
